# Chinese translation validation of the adherence barrier questionnaire (intravitreal anti‐vascular endothelial growth factor therapy) in Taiwan

**DOI:** 10.1002/kjm2.12905

**Published:** 2024-11-14

**Authors:** Yi‐Chun Chi, Hui‐Min Hsieh, Sabrina Müller, Shwu‐Jiuan Sheu

**Affiliations:** ^1^ Department of Ophthalmology Kaohsiung Medical University Hospital Kaohsiung Taiwan; ^2^ Department of Public Health Kaohsiung Medical University Kaohsiung Taiwan; ^3^ Division of Medical Statistics and Bioinformatics, Department of Medical Research Kaohsiung Medical University Hospital Kaohsiung Taiwan; ^4^ Center for Big Data Research Kaohsiung Medical University Kaohsiung Taiwan; ^5^ IPAM e.v, University of Wismar University of Applied Sciences Wismar Germany; ^6^ Department of Ophthalmology, School of Medicine Kaohsiung Medical University Kaohsiung Taiwan

Intravitreal injection (IVI) of anti‐vascular endothelial growth factor (anti‐VEGF) has tremendously changed the management of neovascular age‐related macular degeneration (nAMD) and diabetic macular edema. However, multiple and long‐term injections are required to sustain the therapeutic benefits, which set a large financial and time burden on patients. Medical adherence consequently appears to be crucial for a successful IVI treatment.^1^, ^2^ Attempting to identify the barriers to medical adherence, Müller et al. adapted a questionnaire identifying generalized medical‐related nonadherence,^3^ and introduced a 17‐item instrument for identifying barriers to IVI anti‐VEGF in patients with nAMD and DME, the adherence barrier questionnaire of intravitreal therapy (ABQ‐IVT).^4^ Given no adherence barriers questionnaire of IVI therapy in traditional Chinese, we translated the ABQ‐IVT and evaluated its reliability as well as construct validity.

The ABQ‐IVT was translated by bilingual speakers with forward translation, backward translation, and synthesis process. All 17 translated items were included in the final questionnaire after a preliminary reliability test with Cronbach's alpha coefficient. The final survey was performed from July 2022 to May 2023 in a single medical center in Southern Taiwan under the identical inclusion criteria as the original ABQ‐IVT.^4^

A total of 107 patients (83 with nAMD and 24 with DME) were enrolled. The top impact items on the adherence barrier were Item 10 (time commitment), followed by Item 11 (travel costs) and Item 12 (challenge accompanying person). The least impact item was Item 2 (trust in doctor[s]). The questionnaire was further condensed by excluding five questions from the exploratory factor analysis based on the possibility of duplications, cultural distinctions, or multi‐faceted compositions. After their removal, there were higher loadings for all remaining items, and increased reliability in the new 12‐item questionnaire (Table 1). The exploratory factor analysis suggested a four‐subscale model: burden (burden of finance, time, and family; Items 11, 12, 13, and 15); eye doctor (information, trust, and comfort; Items 1, 2, and 4); treatment (share decision‐making and side effects; Items 3 and 9); individual characteristics (unsatisfactory, depression, and private duties; Items 6, 7, and 16).

**TABLE 1:** Reliability and validity analysis.

Item	Cronbach's alpha	KMO test	Factor 1 (Burden)	Factor 2 (Doctor)	Factor 3 (Treatment)	Factor 4 (Individual)
1. “I generally feel well informed about the treatment of my eye disease”	0.710	0.633		0.754		
2. “I trust my eye doctor(s)”	0.727	0.587		0.848		
3. “My eye doctor includes me in decisions about the course of treatment”	0.712	0.822			0.556	
4. “I often feel uncomfortable in the doctor's office”	0.724	0.760		0.478		
6. “I am dissatisfied with my current care/treatment”	0.741	0.602				0.507
7. “Generally, I often feel downcast and sometimes discouraged and depressed”	0.759	0.423				0.777
9. “I am afraid of the IV treatments and/or the side effects”	0.735	0.684			0.803	
11. “Attending eye doctor appointments poses a high financial burden (e.g., travel costs/absenteeism) for me and/or my relatives”	0.719	0.734	0.680			
12. “Especially doctor's appointments which require an accompanying person pose a challenge”	0.706	0.634	0.890			
13. “I am worried about being a burden to my family/relatives and to have to ask for help”	0.708	0.590	0.897			
15. “Apart from my eye condition I experience other conditions which hampers my attendance to appointments”	0.720	0.847	0.503			
16. “I have private/professional duties which are hardly compatible with the treatment of my eye disease”	0.723	0.804				0.440
Total	0.741	0.674				

Abbreviations: IV, intravitreal; KMO test, Kaiser–Meyer–Olkin test.

To date, this is the first and only validated traditional Chinese questionnaire for assessing the adherence barriers to IVI anti‐VEGF therapy. The most impact adherence barriers in our cohort are similar to those in the original questionnaire from Germany (time commitment, depression, and travel costs).^4^ The top adherence barriers in Turkish were fear of injection, disbelief of the benefit of treatment, financial limitations, and continuation at another center.^5^ Our cohort appears to have better trust in the treatment and less fear in IVI; nevertheless, pose large concerns in financial and time burden. Due to geographic variation in financial capability, educational level, and medical accessibility, along with the growing demand of IVI treatments, there is an urgent need for a localized evaluation for adherence barriers and medical compliance. The present translated questionnaire was proved feasible for IVI patients in Taiwan. Moreover, the reliability increased in the condensed 12‐item questionnaire, which is supposed to make the test easier and more practical in clinical practice.

## CONFLICT OF INTEREST STATEMENT

The authors declare no conflict of interest.
